# Computational Modeling of Open-Irrigated Electrodes for Radiofrequency Cardiac Ablation Including Blood Motion-Saline Flow Interaction

**DOI:** 10.1371/journal.pone.0150356

**Published:** 2016-03-03

**Authors:** Ana González-Suárez, Enrique Berjano, Jose M. Guerra, Luca Gerardo-Giorda

**Affiliations:** 1 BCAM - Basque Center for Applied Mathematics, Bilbao, Basque Country, Spain; 2 Biomedical Synergy, Electronic Engineering Department, Universitat Politècnica de València, València, Spain; 3 Department of Cardiology, Hospital de la Santa Creu i Sant Pau, Barcelona, Spain; Magna Graecia University, ITALY

## Abstract

Radiofrequency catheter ablation (RFCA) is a routine treatment for cardiac arrhythmias. During RFCA, the electrode-tissue interface temperature should be kept below 80°C to avoid thrombus formation. Open-irrigated electrodes facilitate power delivery while keeping low temperatures around the catheter. No computational model of an open-irrigated electrode in endocardial RFCA accounting for both the saline irrigation flow and the blood motion in the cardiac chamber has been proposed yet. We present the first computational model including both effects at once. The model has been validated against existing experimental results. Computational results showed that the surface lesion width and blood temperature are affected by both the electrode design and the irrigation flow rate. Smaller surface lesion widths and blood temperatures are obtained with higher irrigation flow rate, while the lesion depth is not affected by changing the irrigation flow rate. Larger lesions are obtained with increasing power and the electrode-tissue contact. Also, larger lesions are obtained when electrode is placed horizontally. Overall, the computational findings are in close agreement with previous experimental results providing an excellent tool for future catheter research.

## Introduction

Radiofrequency (RF) catheter ablation (RFCA) is a common and safe procedure to eliminate cardiac arrhythmias by means of thermal destruction of the tissue responsible for the arrhythmia. An RF current is delivered via an ablating electrode embedded at the tip of a percutaneous catheter and, as a consequence, a thermal lesion (>50°C) is created in the target tissue. During RFCA, thrombus formation can occur if the electrode-tissue interface temperature reaches around 80°C, resulting in denaturation and adherence of blood proteins [1,2]. In order to prevent this phenomenon and simultaneously achieve larger thermal lesions, open-irrigated electrodes have been developed [3–10]. These catheters allow continuous saline flushing at the blood-tissue interface through small holes distributed around the electrode tip [3,7].

Computer modeling has been used in numerous studies on RFCA [11–17]. This approach allows to study different issues involved in RFCA in an efficient and reliable way, as opposed to *ex vivo*-*in vitro* and *in vivo* setups, which are time-consuming and lack reproducibility due to the several variables involved. To date, all previous models for endocardial RFCA mimicked the effect of the saline irrigation by simply fixing a constant temperature in the electrode tip, i.e. ignoring the saline motion problem [6,11]. The challenge of a realistic model of open-irrigated electrode is to account for both the saline irrigation flow and the blood flow motion.

The previous models did not solve the fluid motion problem associated with the circulating blood, and modeled the thermal effect by considering a convective boundary condition at the blood-tissue interface. This approach allows to adequately predict the lesion depth created in the myocardial tissue, but unfortunately it does not allow to obtain a realistic distribution of blood temperature in the cardiac chamber [12,17]. As commented, the blood temperature reached around the tip of the electrode is crucial since an excessive temperature directly leads to thrombus formation. To our knowledge, there is only one computational model of open-irrigated catheter that accounts for the saline irrigation flow, but it focuses on epicardial RF ablation and hence the circulating blood does not interact with the saline irrigation [13]. Our objective was to set up the first computer model for open-irrigated electrode in endocardial RFCA which takes into account simultaneously the saline irrigation and the blood motion. Once the model was built, we conducted computer simulations under different conditions of irrigation flow rate, electrode-tissue contact and power settings. The thermal performance of the model was validated by comparing the computer results with previous experimental studies.

## Materials and Methods

### Description of the model geometry

**Fig 1:**
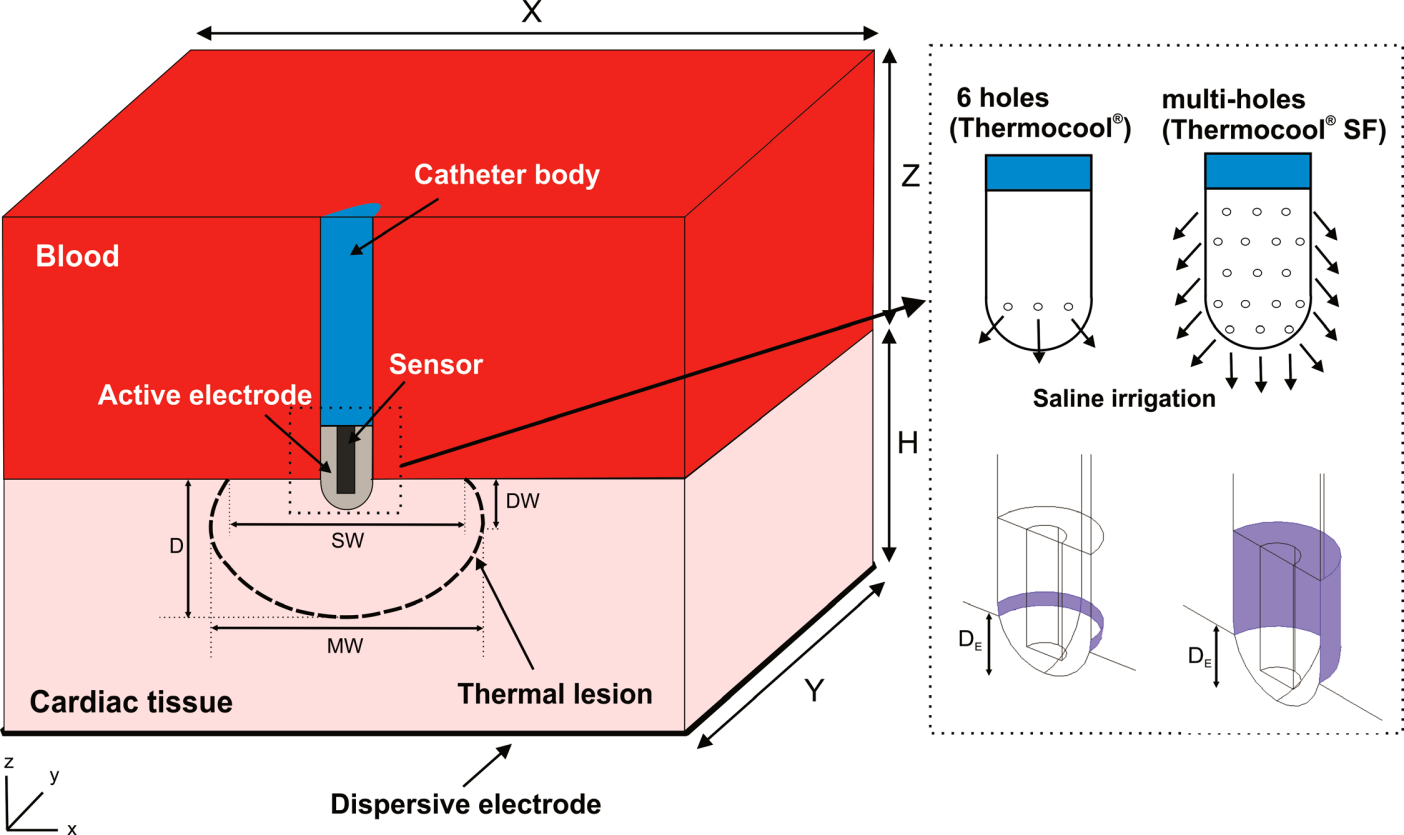
Geometry of the computational model built (figure not to scale). Note that XZ-plane is the symmetry plane in the model. Cardiac tissue thickness (H) is 20 mm [4,12], while the dimensions of the fragment of cardiac chamber of X = 80 mm and Y = 40 mm (Z = Y) are estimated by means of a convergence test. Two active electrodes (8Fr, 3.5 mm) are considered (right image): 6-holes and multi-holes open-irrigated electrodes as models ThermoCool^®^ and ThermoCool^®^ SF (Biosense Webster, Diamond Bar, CA, USA), respectively. These are inserted into cardiac tissue a depth (D_E_) of 1 mm. The saline irrigation through the small holes in the electrode tip is modeled by an inlet velocity boundary condition at the electrode-blood interface. Thermal lesion is assessed by the 50°C isotherm and its geometry is characterized by: maximum depth (D), maximum width (MW), depth at the maximum width (DW), and surface width (SW).

Fig 1 shows the geometry and dimensions of the three-dimensional computational model, where the XZ-plane is the symmetry plane. The model consists of a fragment of cardiac tissue and a volume of blood (cardiac chamber). The open-irrigated electrode is initially in perpendicular position with respect to the cardiac tissue [3]. The electrode includes a sensor embedded within its tip, which is used for monitoring the electrode temperature [3,7–9]. The dispersive electrode is modeled as an electrical boundary condition at a distance from the active electrode. The cardiac chamber dimensions (X, Y and Z) are estimated by means of a convergence test in order to avoid boundary effects. In this test, the value of the maximal temperature achieved in the tissue (T_max_) after 60 s of RF heating is used as control parameter. We first considered a tentative spatial (i.e. minimum meshing size) and temporal resolution. To determine the appropriate parameters of X and Y (Z = Y), we increased their values by equal amounts. When the difference in the T_max_ between consecutive simulations was less than 0.5%, we considered the former values to be adequate. We then determined adequate spatial and temporal resolution by means of similar convergence tests using the same control parameter as in the previous test. Discretization is spatially heterogeneous: the finest zone is the electrode-tissue interface, where the largest voltage gradient is produced and hence the maximum value of current density. In the tissue, grid size increases gradually with distance from the electrode-tissue interface.

We considered two designs of open-irrigated electrode (8Fr diameter and 3.5 mm length) with 6- and multi-holes in the distal zone, which correspond with two commercial models for RFCA: ThermoCool^®^ and ThermoCool^®^ SF (Biosense Webster, Diamond Bar, CA, USA), respectively. The small holes allow the saline solution to continuously flush into the cardiac chamber. In endocardial ablation, the flowing saline mixes with the circulating blood without forming a film around the electrode, differently from what happens in epicardial ablation [13]. Since the blood and the saline feature comparable densities, the mixing can be considered to be homogeneous, and consequently the effect of saline flow can be modeled as an inlet boundary condition into the blood region, specifically a condition set at the zone where the holes are located. To reduce computational complexity, the irrigation holes are not individually included in the physical model, as their tiny sizes would require an unnecessarily fine computational mesh in their neighborhood. In this way, the inlet boundary condition to account for the effect of saline irrigation is imposed on the zone of the electrode surface corresponding with the holes location (see violet surface in Fig 1). We assume that a saline film is not forming at the electrode-tissue interface since the tissue presses the holes in this zone and blocks the saline irrigation flow out. As a consequence, a boundary condition associated with the saline irrigation need not being included at the semispherical part of the electrode tip inserted in the tissue. The electrode similar to ThermoCool^®^ features 6 holes arranged 60° apart from each other on a section of its distal tip which is perpendicular to the electrode axis. Therefore, the inlet boundary condition is applied to a ring on the electrode-blood surface of 1.32 or 0.77 mm^2^ in case of that the electrode is placed in perpendicular or parallel position with respect to the tissue. In contrast, the multi-holes electrode as ThermoCool^®^ SF features 56 holes distributed around its entire distal tip, thus the inlet boundary condition is applied to the whole electrode-blood surface above the tissue of 10.37 mm^2^ when the electrode is in perpendicular position (Fig 1) or to a surface of 8.58 mm^2^ whether the electrode is placed in parallel position.

### Governing Equations

The numerical model is based on a coupled electric-thermal-flow problem, which is solved numerically using the Finite Element Method (FEM) with COMSOL Multiphysics software (COMSOL, Burlington, MA, USA). The governing equation for the thermal problem is the *Bioheat Equation*, modified by the enthalpy method [18] that includes the phase change to model tissue vaporization:

$$\frac{\partial \left(\rho h\right)}{\partial t}=\nabla \cdot (k\nabla T)+q-Q_{p}+Q_{m}-\rho cu\cdot \nabla T$$ (1)

where *ρ* is density (kg m^-3^), *h* enthalpy, *t* time (s), *k* thermal conductivity (W m^-1^ K^-1^), *T* temperature (°C), *q* the heat source caused by RF power (W m^-3^), *Q*_*p*_ the heat loss caused by blood perfusion (W m^-3^) and *Q*_*m*_ the metabolic heat generation (W m^-3^). *Q*_*p*_ can be neglected since it has been demonstrated both computationally [19] and experimentally [20] that the blood flow away from the coronary arteries does not have significant influence on the temperature distribution during RFCA. Also, *Q*_*m*_ is not considered because it is negligible in comparison to the other terms [14]. In biological tissues, enthalpy is related to temperature by the following expression [18]:

$$\frac{\partial (\rho h)}{\partial t}=\frac{\partial T}{\partial t}\cdot \left\{ \begin{array}{l} \rho_{l}c_{l}\;0\leq T\leq 99{}^{\circ}C\\ H_{fg}C\;99<T\leq 100{}^{\circ}C\\ \rho_{g}c_{g}\;T>100{}^{\circ}C \end{array}\right.$$ (2)

where *ρ*_*i*_ and *c*_*i*_ are the density and specific heat of cardiac tissue before phase-change (i.e. liquid phase, *i = l*) and post-phase-change (i.e. gas phase, *i = g*), respectively; *H*_*fg*_ is the latent heat and *C* the tissue water content. We considered a latent heat value of 2.162×10^9^ J m^-3^ which corresponds to the product of the water vaporization latent heat (2257 kJ kg^-1^), the water density at 100°C (958 kg m^-3^), and the tissue water content inside the cardiac tissue (75%).

The last term in the Eq (1) corresponds to the advection term [12], which represents the heat loss due to blood flow. The velocity field ***u*** = (***u***_*x*_, ***u***_*y*_, ***u***_*z*_) (m/s) is described by *the incompressible Navier-Stokes Equations* of fluid dynamics, consisting of a Momentum equation and a Mass equation:

$$\rho \frac{\partial u}{\partial t}+\rho u\cdot \nabla u=-\nabla P+\mu \nabla^{2}u+F$$ (3)

∇⋅u=0 (4)

where *P* is the pressure (Pa), *μ* is the viscosity (for blood 2.1×10^−3^ kg m^-1^ s^-1^) [17], while ***F*** = (*F*_*x*_, *F*_*y*_, *F*_*z*_) represents body forces (N m^-3^), neglected in our model.

At frequencies used in RF heating (≈500 kHz) and over the distance of interest, the biological medium can be considered almost totally resistive, and a quasi-static approach can therefore be used to solve the electrical problem [21,22]. The distributed heat source *q* is then given by *q = σ|****E****|*^*2*^, where *|****E****|* is the magnitude of the vector electric field (V/m) and *σ* the electrical conductivity (S m^-1^). ***E*** = −∇Ф is calculated from the gradient of the voltage Φ (V), which, in absence of internal electric sources, satisfies ∇·(*σ*∇Ф) = 0.

### Properties of the model elements

The thermal and electrical properties of the model elements are shown in Table 1 [17]. The initial model temperature is 37°C, except in the electrode tip which is 22°C, imposed as a consequence of the saline irrigation [23]. The electrical (*σ*) and thermal conductivity (*k*) of cardiac tissue are temperature-dependent piecewise functions defined as:

$$\sigma (T)=\left\{ \begin{array}{ll} \sigma_{o}e^{0.015(T-37)} & \;0\leq T\leq 100{}^{\circ}C\\ \sigma_{o}\cdot 2.5728-\sigma_{o}\cdot 0.5145(T-100) & \;100\leq T\leq 105{}^{\circ}C\\ \sigma_{o}\cdot 2.5728\cdot 10^{-4} & \;T>105{}^{\circ}C \end{array}\right.$$ (5)

$$k(T)=\left\{ \begin{array}{l} k_{o}+0.0012(T-37)\;0\leq T\leq 100{}^{\circ}C\\ k_{o}+0.0756\;T>100{}^{\circ}C \end{array}\right.$$ (6)

**Table 1:** Thermal and electrical characteristics of the elements employed in the numerical models (data from [17]): *σ*: electric conductivity; *k*: thermal conductivity; *ρ*: density; *c*: specific heat.

Element/Material		*σ* (S m^-1^)	*k* (W m^-1^K^-1^)	*ρ* (kg m^-3^)	*c* (J kg^-1^K^-1^)
Electrode/Pt-Ir		4.6×10^6^	71	21500	132
Thermistor/Glass fiber		10^−5^	0.038	32	835
Catheter/Polyurethane		10^−5^	0.026	70	1045
Cardiac chamber/Blood		0.667	0.54	1000	4180
Myocardium/Cardiac tissue	Liquid phase	*σ*_o_*	*k*_o_*	1060	3111
	Gas phase			370.44	2155.92

*σ_*o*_ and *k*_*o*_ were assessed at 37°C. *k*_*o*_ = 0.531 [17] and *σ*_*o*_ was adjusted to achieve the same initial impedance as in the *in vitro* setup (≈ 140 Ω) [3]: 0.12 S m^-1^.

The electrical conductivity *σ* features, between 0 and 100°C an exponential growth of 1.5°C^-1^ [14], a linear decay of 4 orders of magnitude for 5°C to model the tissue desiccation [24], and then it remains constant. In Eq 5, *σ*_*o*_ is the value of the electrical conductivity assessed at 37°C. In order to compare our computational results with a previous experimental study [3], the value of *σ*_*o*_ was adjusted to obtain the same initial impedance as in the experiments (≈ 140 Ω). The thermal conductivity *k* (Eq 6) grows linearly 0.12°C^-1^ up to 100°C, *k*_*o*_ being the value of *k* assessed at 37°C (see Table 1), and then it is kept constant [22].

### Boundary conditions

**Fig 2:**
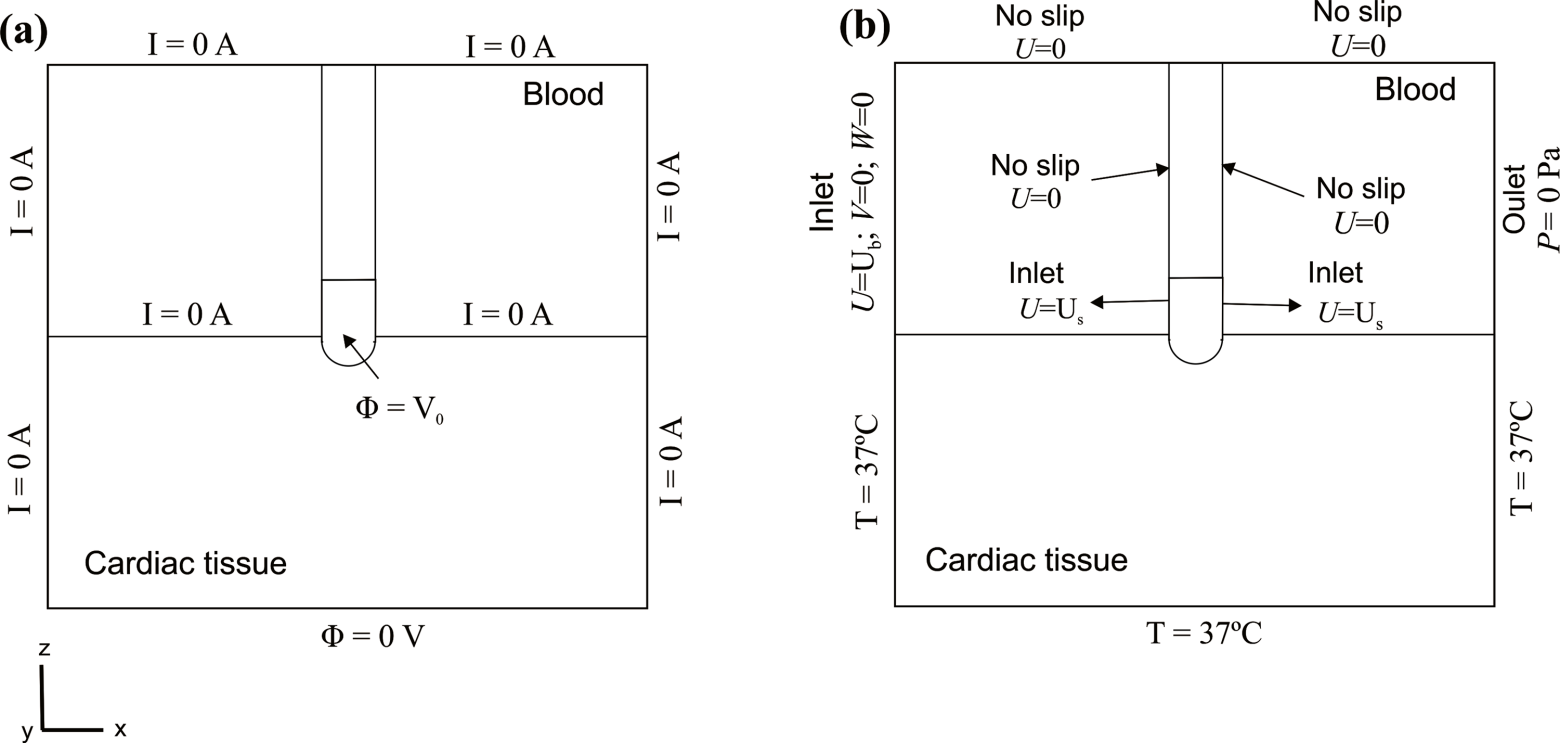
**Electrical (a) and thermal and velocity (b) boundary conditions of the model.** Note that the effect of saline irrigation is simulated as an inlet velocity boundary condition into the blood region, since the irrigation holes are not included in the model and the saline irrigation is not considered inside the electrode.

Fig 2A shows the electrical boundary conditions. We consider a constant power ablation, which is the usual ablation mode for open-irrigated electrodes in RFCA. We implemented a proportional-integral (PI) control algorithm using MATLAB (MathWorks, Natick, MA, USA) and is simulated by means of the COMSOL-MATLAB interface, where we previously exported the FEM structure generated from COMSOL. The behavior of the PI controller is determined by the proportional (*K*_*p*_) and the integration (*K*_*i*_) constant. We used the parameters for a standard PI controller *K*_*p*_ = 4.78, *K*_*i*_ = 3.39. The output of the PI controller corresponds to the applied voltage, which is modulated to maintain the delivering power within 3% of the target [25]. Therefore, a voltage boundary condition is applied at the active electrode surface. On all the outer surfaces of the model, except the bottom surface, a null electrical flux is imposed. The voltage on the bottom surface is set to 0 V to model the dispersive electrode.

Fig 2B shows the thermal and velocity boundary conditions. For the thermal boundary conditions, a null thermal flux is used on the symmetry plane and a constant temperature of 37°C is fixed on the outer model surfaces. For the velocity boundary conditions, an inlet velocity boundary condition is applied on the left surface of the fluid volume to impose a blood flow velocity (in *x*-direction) of 0.1 m s^-1^ [3,7–9]. An outlet boundary condition of zero pressure is fixed on the right surface of the fluid volume. The saline irrigation flow is taken into account by an inlet velocity condition into the blood region, applied to a specific part of the electrode-blood interface surface, and calculated as the ratio between the saline irrigation flow rate (Q) and the electrode area though which the saline flows (see violet surface in Fig 1). A no slip condition is applied on the upper surfaces of the fluid volume, at the symmetry plane and at the tissue-blood and electrode-blood interfaces.

### Lesion assessment

The thermal lesions are identified by the 50°C isotherm contour as in previous studies [14–17,25], which is usually considered to reasonably represent the isotherm of irreversible myocardial injury in hyperthermic ablation. The thermal lesions are determined by their characteristic dimensions (see Fig 1) [3,7,8,22,26]: maximum depth (D), maximum width (MW), depth at the maximum width (DW), and surface width (SW). We assessed the lesion volume (LV) using the formula described in [3,5,27].

### Validation of the model and computer simulations

We assess the accuracy of the computational model by comparing the lesion dimensions obtained for both electrode designs with available data from experiments with ThermoCool^®^ (6-holes electrode) and ThermoCool^®^ SF (multi-holes electrode) [3]. In brief, these experiments were conducted using constant power of 20 and 35 W for 30 and 60 s with the electrode in perpendicular position respect to the tissue. After RF energy, the myocardium was cross-sectioned at the level of each lesion and stained with TTC (triphenyltetrazolium chloride) to evaluate the thermal lesion. We set the saline irrigation flow rate to 13 mL min^-1^ as in these experiments, so that the saline flow velocity applied on the electrode surface is 0.164 and 0.021 m s^-1^ for the 6-holes and multi-holes electrode, respectively. We consider that the differences in D and MW are negligible for values less than 1 mm, since this value is approximately that of the deviation (±0.5 mm) observed in experimental RFCA studies [4,28].

Once we checked that the computational model predicts reasonably well the experimental lesions, we studied the variations in both the lesion dimensions created in the tissue and the maximum blood temperature attained in the cardiac chamber (T_max_blood_) for different configurations, considering both electrode designs (6-holes and multi-holes) and two electrode positions respect to the tissue surface (perpendicular and parallel). First, we assessed the effect of varying the saline irrigation flow rate from 5 to 20 mL min^-1^ [10]. Then, we studied the impact of changes in the applied power and the electrode-tissue contact pressure. In these last simulations we used the irrigation flow rate recommended by the manufacturer of the commercial electrodes (ThermoCool^®^ and ThermoCool^®^ SF) according to the RF power applied [26]. In the case of the 6-holes electrode (ThermoCool^®^), the recommended flow rate is 17 mL min^-1^ for RF power levels below 30 W, and 30 mL min^-1^ for RF power between 31 W and 50 W. For the multi-holes electrode (ThermoCool^®^ SF), the recommended flow rate is 8 mL min^-1^ for RF power levels below 30 W, and 15 mL min^-1^ for RF power between 31 W and 50 W.

## Results

### Validation of the computational model

The convergence tests provided a optimal chamber dimensions of X = 80 mm and Y = 40 mm (Z = Y), a grid size of 0.2 mm in the finest zone (electrode-tissue interface), and a time step of 0.05 s.

**Fig 3:**
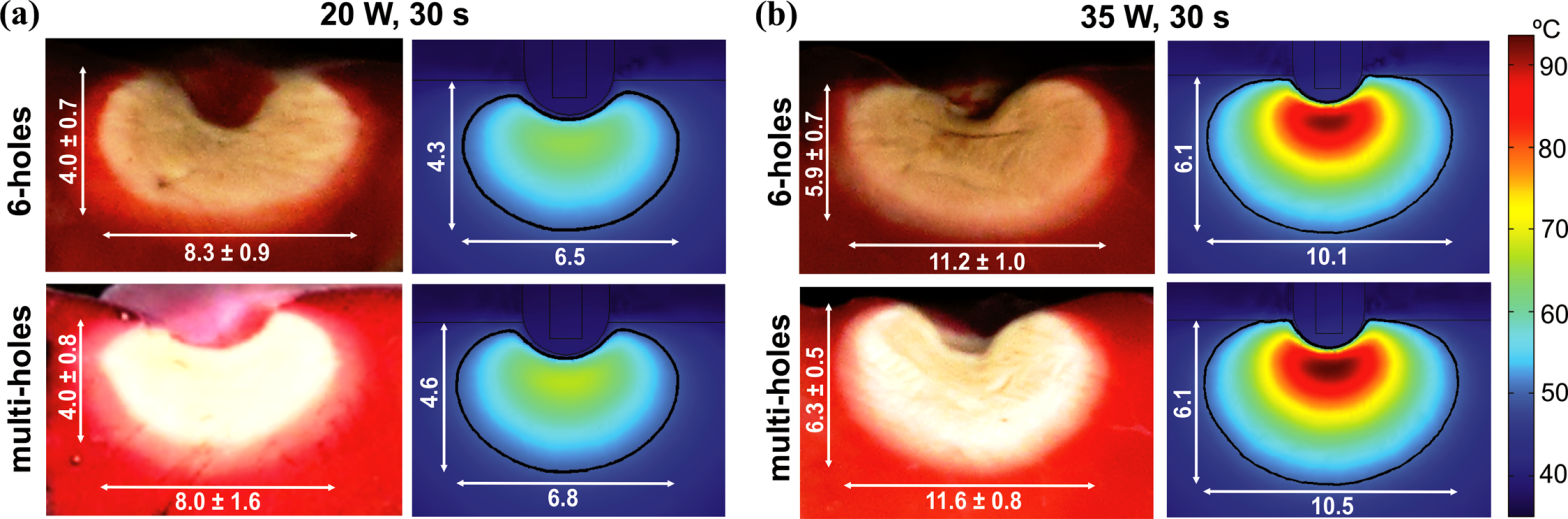
Validation of the computational model. Experimental (data from [3]) and computational thermal lesions created in the tissue after 30 s of RFCA using power-control mode of 20 and 35 W. The thermal lesion contour was assessed by the central "white zone" in the experiments and by the 50°C isotherm in the computational model.

In Fig 3 we compare experimental and computational thermal lesions created in the tissue after 30 s of RFCA using power control-mode of 20 W and 35 W for both open-irrigated electrodes. The thermal lesion contour in experiments was assessed by the central "white zone" excluding the surrounding pale-pink zone, which was considered as non-coagulated tissue (viable tissue). The experimental and computational thermal lesion shapes show a close agreement. Table 2 shows the lesion dimensions (D and MW) obtained from experiments and numerical simulations after 30 or 60 s of RFA using 20 and 35 W for both designs of open-irrigated electrodes. The depths obtained from simulations are in general very similar to the experimental results for both electrode designs (differences below 1 mm). The only exception appears when a power of 35 W is applied for 60 s, where D is slightly larger than the experimental ones (1 mm and 1.2 mm bigger for the 6-holes and multi-holes electrode, respectively). About MW, computer simulations provide in general values of MW 1–2 mm smaller with respect to experimental results. In spite of this, the model shows a good thermal performance respect to the applied power and duration. In fact, the experimental and computational differences in MW, when the applied power and duration are changed, are always within 1 mm.

**Table 2:** Lesion dimensions (maximum depth D, and maximum width MW) from the experimental [3] and the computational model after 30 or 60 s of RFCA using power-control mode of 20 and 35 W, with 6-holes and multi-holes open-irrigated electrodes as models ThermoCool and ThermoCool SF in [3]. The electrode was in perpendicular position respect to the cardiac tissue and the irrigation flow rate was 13 mL min^-1^.

		Experimental		Computational	
	P (W), t (s)	6-holes	multi-holes	6-holes	multi-holes
D (mm)	20 W, 30 s	4.0 ± 0.7	4.0 ± 0.8	4.3	4.6
	20 W, 60 s	5.4 ± 0.9	5.4 ± 0.8	5.6	5.9
	35 W, 30 s	5.9 ± 0.7	6.3 ± 0.5	6.1	6.1
	35 W, 60 s	7.2 ± 0.8	7.0 ± 0.3	8.2	8.2
MW (mm)	20 W, 30 s	8.3 ± 0.9	8.0 ± 1.6	6.5	6.8
	20 W, 60 s	10.1 ± 1.6	9.9 ± 1.8	7.9	8.3
	35 W, 30 s	11.2 ± 1.0	11.6 ± 0.8	10.1	10.5
	35 W, 60 s	13.5 ± 1.7	12.9 ± 1.7	11.9	12.1

### Impact of the irrigation flow rate

**Fig 4:**
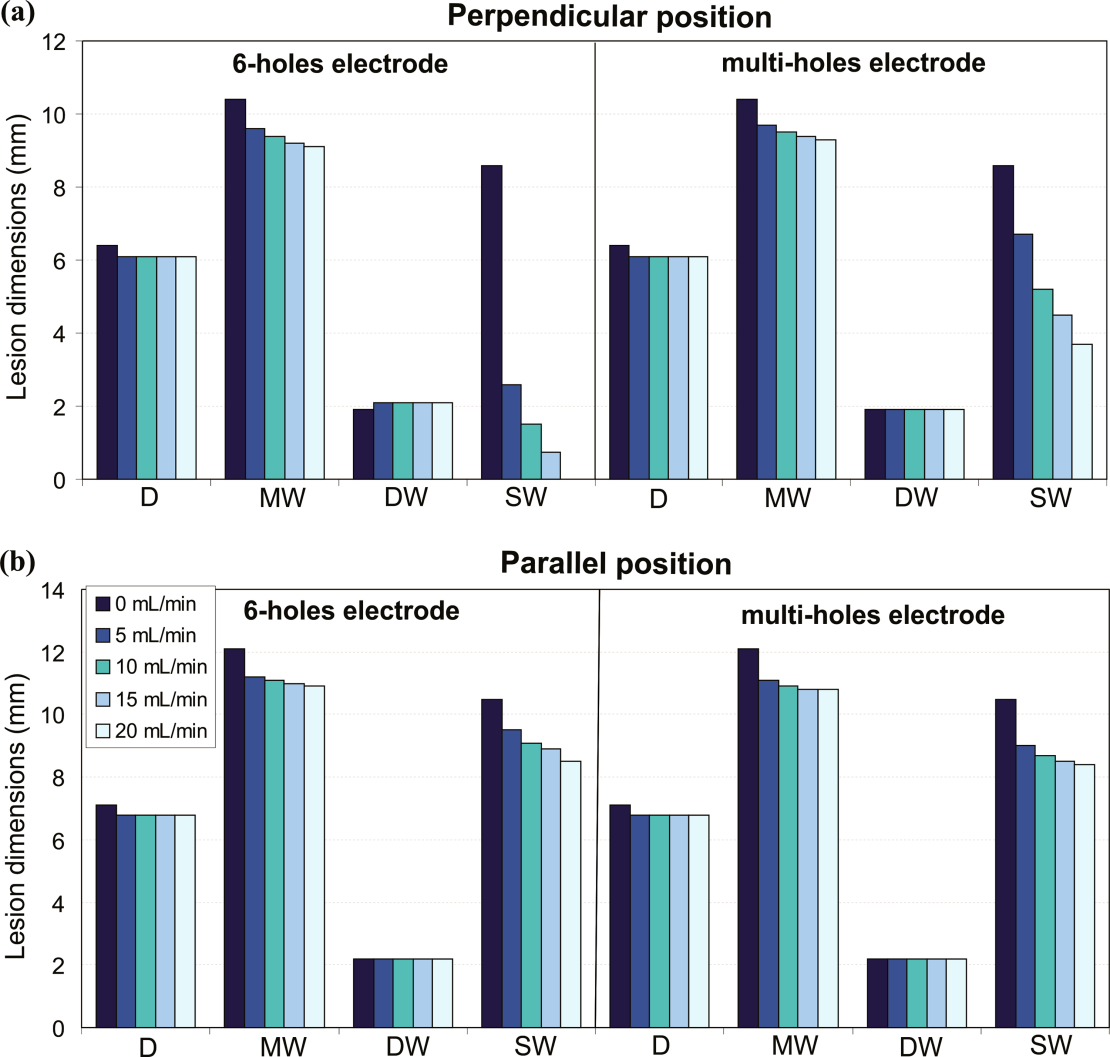
**Effect of the irrigation flow rate**: lesion dimensions (D: maximum depth, MW: maximum width, DW: depth at the maximum width, and SW: surface width) obtained after 30 s of RFA with power-control mode of 35 W, considering four irrigation flow rates (5, 10, 15 and 20 mL min^-1^), two designs of open-irrigated electrode tip (6-holes and multi-holes), and perpendicular and parallel electrode-tissue positions.

Fig 4 shows the effect of changing the irrigation flow rate (from 5 mL min^-1^ to 20 mL min^-1^) on the lesion dimensions after 30 s of RFCA with power-control mode of 35 W for both electrode designs and positions. For the sake of completeness, we include also the case of 0 mL min^-1^, which represents a non-irrigated electrode. Neither of the two open-irrigated electrodes shows an impact on D and DW, when the irrigation rate is changed. Comparing non-irrigated and irrigated electrodes, D is slightly larger with non-irrigated electrode in all cases: D was 6.4 mm and 6.1 mm in perpendicular position and 7.1 mm and 6.8 mm in parallel position for non-irrigated and irrigated electrodes, respectively. In contrast, MW and SW are smaller when irrigation flow rate is increased, regardless of the electrode design and position. The reduction of SW is particularly pronounced when the irrigation flow rate is increased, especially for the 6-holes electrode in perpendicular position (Fig 4A): SW is already drastically reduced from 8.6 mm of a non-irrigated electrode to 2.6 mm for an electrode with 5 mL min^-1^ irrigation flow rate; and increasing the flow rate, SW is progressively reduced to 1.5 mm for 10 mL min^-1^ and to 0 mm for 20 mL min^-1^.

**Fig 5:**
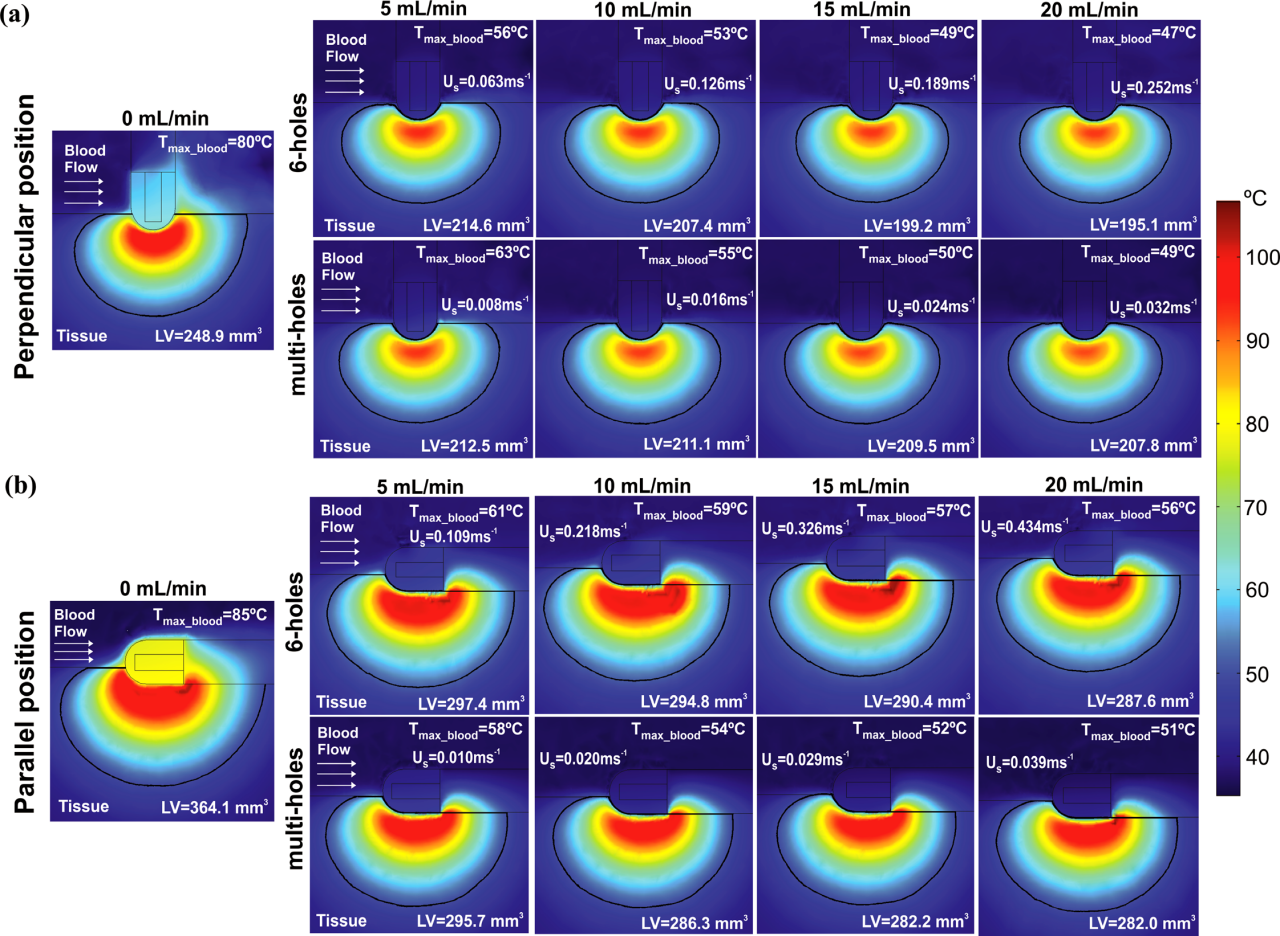
**Effect of the irrigation flow rate**: temperature distributions in the cardiac tissue and blood and lesion volume (LV) after 30 s of RFCA with power-control mode of 35 W, considering four irrigation flow rates (5, 10, 15 and 20 mL min^-1^), two designs of open-irrigated electrode tip (6-holes and multi-holes), and perpendicular (a) and parallel (b) electrode-tissue positions. The solid black line corresponds to the 50°C isotherm. Note that the two left plots (0 mL min^-1^) represent the cases without saline irrigation (non-irrigated). The figure shows the saline flow velocity (U_s_) applied on the electrode surface in each case.

Fig 5 shows the lesion volume (LV) and the temperature distributions in the cardiac tissue and blood. LV is also reduced with the increase of the irrigation flow rate for all cases, more evidently for the 6-holes electrode in perpendicular position: LV decreases from 248.9 mm^3^ to 195.1 mm^3^ when the irrigation flow is incremented from 0 to 20 mL min^-1^. T_max_blood_ decreases as the irrigation flow rate is increased for both electrode designs and positions. The only situation where a critical value of T_max_blood_ above 80°C was registered, hinting the possible thrombus formation at the electrode surface, occurs for the non-irrigated electrode (80°C and 85°C, in perpendicular and parallel position, respectively). On the contrary, T_max_blood_ always stays below 63°C for open-irrigated electrodes. Once again, as for SW, the lowest value of T_max_blood_ is attained by the 6-holes electrode in perpendicular position, for all irrigation flow rates: T_max_blood_ drops by 9°C when the irrigation flow rate is increased from 5 to 20 mL min^-1^. In fact, a strong correlation exists between SW and T_max_blood_ for each electrode design and position: the coefficient of determination R^2^ is always above 0.88, and becomes particularly remarkable for the 6-holes electrode in perpendicular position (R^2^ = 0.99).

### Impact of the electrode design

Only SW is affected by the electrode design when the electrode is placed in perpendicular position. As Fig 4A shows, the 6-holes electrode features smaller values of SW than the multi-holes electrode for all irrigation flow rates: under a flow rate of 5 mL min^-1^, a SW of 2.6 mm and 6.7 mm is obtained with the 6-holes electrode and the multi-holes electrode, respectively. This evident difference in SW, however, does not yield important differences in LV (Fig 5): 212.5 mm^3^ and 214.6 mm^3^ for the 6-holes and multi-holes electrodes, respectively, in perpendicular position with an irrigation flow rate of 5 mL min^-1^. With perpendicular position, the 6-holes electrode features lower values of T_max_blood_ than the multi-holes electrode, independently of the irrigation flow rate. However, with parallel position, the reverse is occurring, and lower values of T_max_blood_ are associated with the multi-holes electrode (see Fig 5).

**Fig 6:**
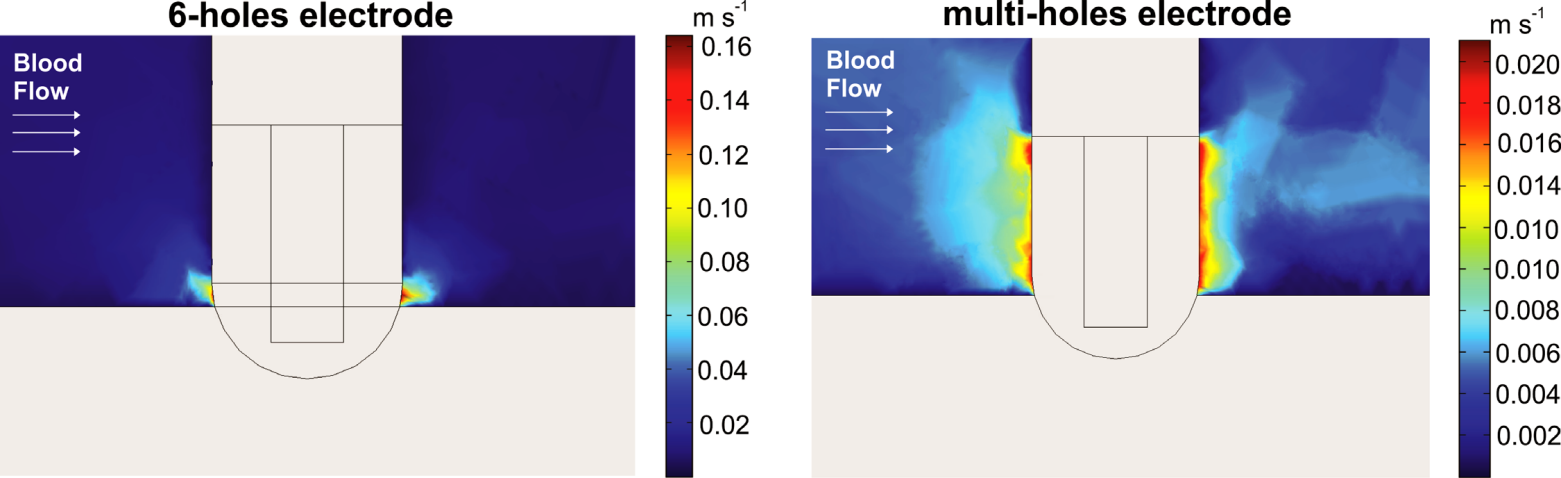
Velocity field distributions for the 6-holes and multi-holes electrode with the electrode in perpendicular position respect to the tissue and considering an irrigation flow rate of 13 mL min^-1^.

Fig 6 shows the velocity field distributions for the two electrode designs. Note that the velocity is much higher around the 6-holes electrode due to this design has a smaller irrigation effective area. Interestingly, velocity distributions were more or less uniform around the electrode, which suggests that the blood velocity has little impact on the velocity distribution of the infused saline next to the electrode surface.

### Impact of the electrode position

Both MW and SW are larger with the electrode in parallel position, for any electrode design (Fig 4): for the 6-holes electrode with a flow of 5 mL min^-1^, MW and SW are incremented by 1.6 mm and 6.9 mm, respectively, when the electrode is in parallel position. In contrast, depth is not importantly affected by the electrode position since its variation is just 0.7 mm. LV is also bigger with the electrode in parallel position, independently from the electrode design (see Fig 5): 214.6 mm^3^ and 297.4 mm^3^ for the 6-holes electrode in perpendicular and parallel position respectively, with a flow rate of 5 mL min^-1^.

The T_max_blood_ is also affected by the electrode position. For the 6-holes electrode, the value of T_max_blood_ is higher with the electrode in parallel position. The difference in the T_max_blood_ between parallel and perpendicular positions grows with the irrigation flow rate: from 5°C (5 mL min^-1^), to 6°C (10 mL min^-1^), 8°C (15 mL min^-1^) and 9°C (20 mL min^-1^). For the multi-holes electrode, on the other hand, T_max_blood_ is higher with the electrode in perpendicular position for lower irrigation flow rates, and the other way around for higher flow rates.

### Impact of the applied power

**Fig 7:**
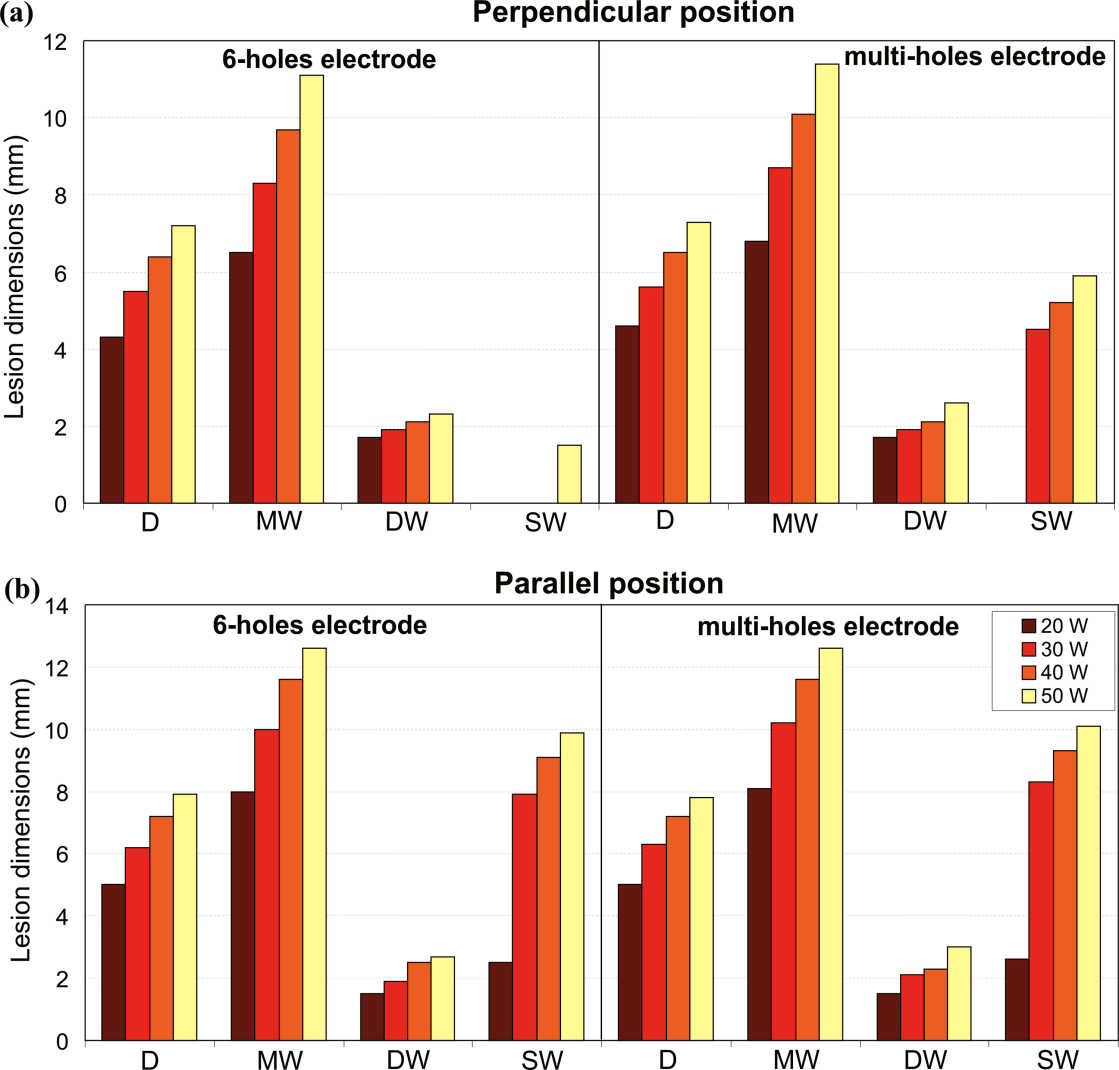
**Effect of the applied power**: lesion dimensions (D: maximum depth, MW: maximum width, DW: depth at the maximum width, and SW: surface width) obtained after 30 s of RFCA with power-control mode at 20, 30, 40 and 50 W, considering perpendicular and parallel electrode-tissue positions, and two designs of open-irrigated electrode tip: 6-holes (a) and multi-holes (b).

Fig 7 shows the effect of variations in the applied power on the lesion dimensions after 30 s of RFCA with power-control mode between 20 W and 50 W. All the parameters characterizing the lesion grow with the increase of the applied power, independently of the electrode design and position. All these parameters grow in a progressive manner with the increase of the applied power, except for SW. The behavior of SW depends on the electrode position, and also on the electrode design when the electrode is in perpendicular position. The value of SW is always larger in parallel than in perpendicular position for any applied power and both electrode designs. For the 6-holes electrode in parallel position, SW abruptly jumps from 2.5 mm to 7.9 mm when the power is increased from 20 W to 30 W, and then it progressively grows up to 9.9 mm at 50 W. When the electrode is in perpendicular position, SW is absent for both designs with the lowest applied power (20 W), while significant differences appear at higher applied power. For the multi-holes electrode, a SW of 4.5 mm appears at 30 W and gradually grows up to 5.9 mm at 50 W. For the 6-holes electrode, SW is absent until the applied power reaches 50 W, where a SW of 1.5 mm appears.

**Fig 8:**
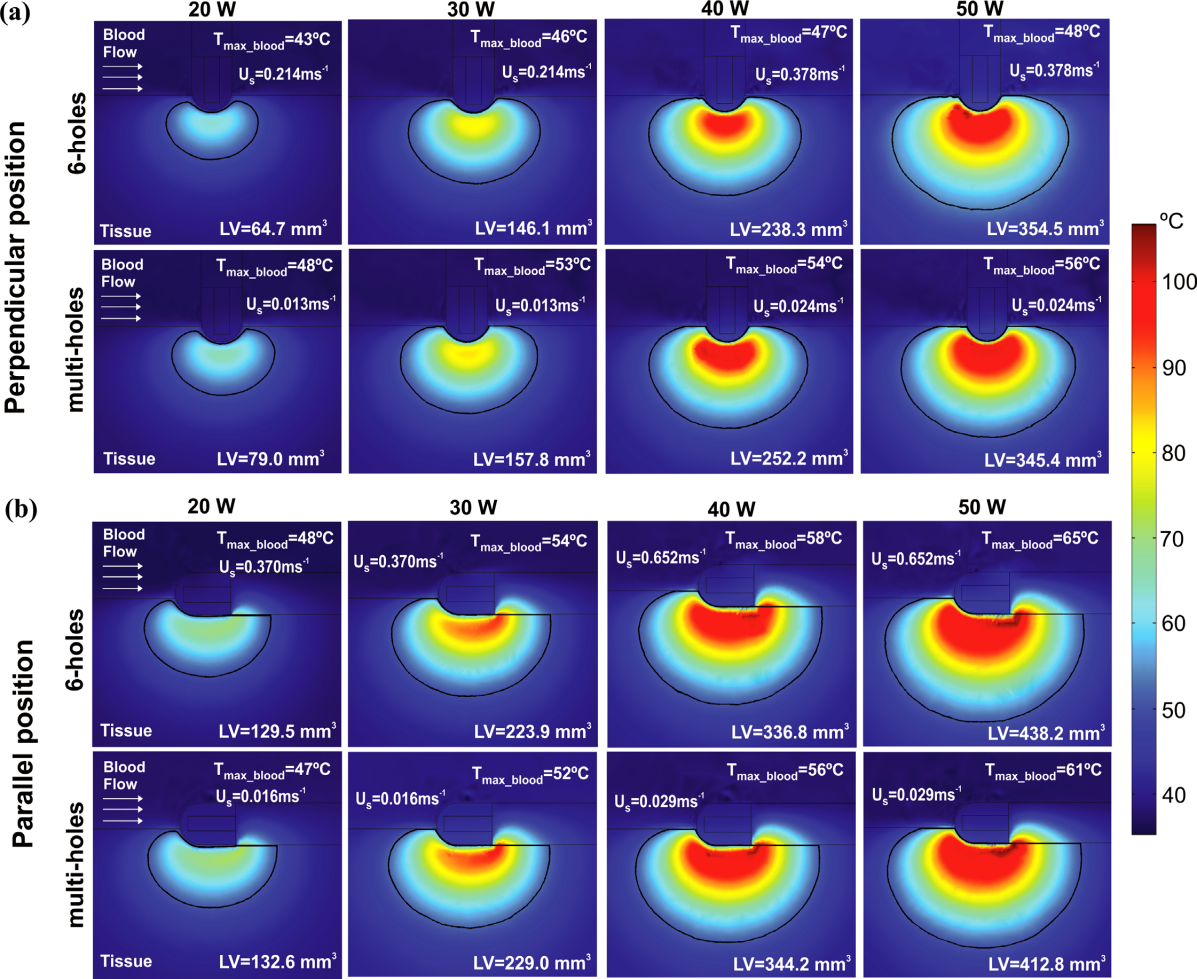
**Effect of the applied power**: temperature distributions in the cardiac tissue and blood and lesion volume (LV) after 30 s of RFCA with power-control mode at 20, 30, 40 and 50 W, considering two designs of open-irrigated electrode tip (6-holes and multi-holes), and perpendicular (a) and parallel (b) electrode-tissue positions. The solid black line corresponds to the 50°C isotherm. The figure shows the saline flow velocity (U_s_) applied on the electrode surface in each case.

Fig 8 shows the corresponding LV and the temperature distributions in the cardiac tissue and blood. As expected, LV grows with the increase of the applied power for any electrode design and position. This increment is larger with the 6-holes than the multi-holes electrode for both electrode positions: LV for the 6-holes electrode increased by 289.8 mm^3^ in perpendicular position and by 308.7 mm^3^ in parallel position when the applied power was increased from 20 W to 50 W. For the multi-holes electrode, the corresponding increment is of 266.4 mm^3^ in perpendicular position, and of 280.2 mm^3^ in parallel position. Higher applied powers yield higher values of T_max_blood_, for any electrode design and position. This increment is higher when the electrode is in parallel position independently of the electrode design, with a larger variation for the 6-holes electrode. In the specific case, when the applied power is increased from 20 W to 50 W, T_max_blood_ increases by 17°C for the 6-holes electrode, and by 14°C for the multi-holes electrode. It is important to note that the value of T_max_blood_ stays below 65°C in all the cases, thus the critical value of T_max_blood_ of 80°C that could suggest thrombus formation was not reached in any situation.

### Impact of the electrode-tissue contact pressure

**Fig 9:**
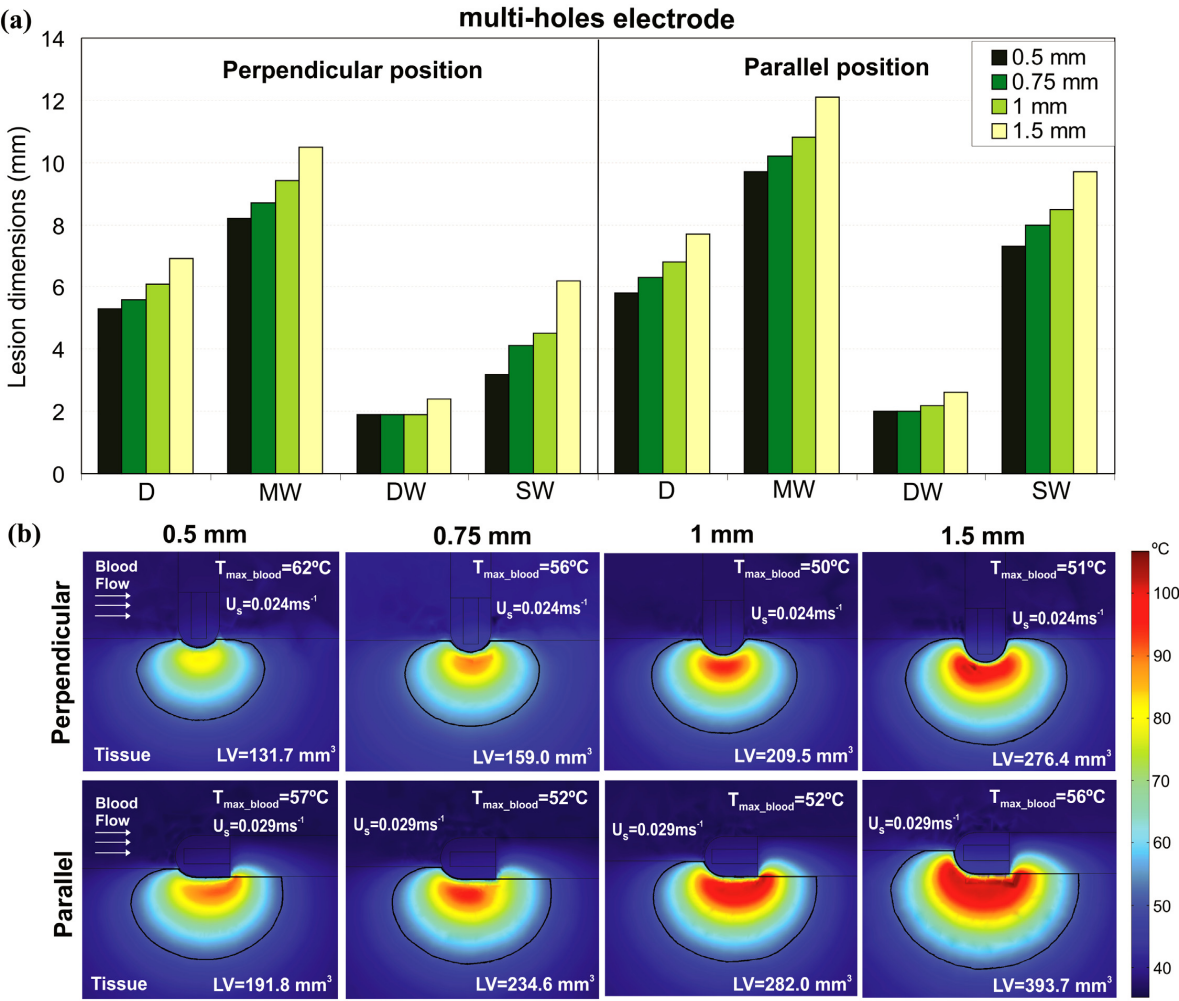
**Effect of the electrode-tissue contact pressure**: (a) lesion dimensions (D: maximum depth, MW: maximum width, DW: depth at the maximum width, and SW: surface width) and (b) temperature distributions in the cardiac tissue and blood and lesion volume (LV) obtained with the multi-holes open-irrigated electrode after 30 s of RFCA considering power-control mode of 35 W (the irrigation flow rate recommended by manufacturer is 15 mL min^-1^), different electrode-tissue contact pressures (0.5, 0.75, 1 and 1.5 mm), and perpendicular and parallel electrode-tissue positions. The solid black line corresponds to the 50°C isotherm. The figure shows the saline flow velocity (Us) applied on the electrode surface in each case.

Fig 9A shows the effect of increasing the electrode-tissue contact pressure on the lesion dimensions after 30 s of RFCA with a power-control mode of 35 W using the multi-holes electrode. The change in contact pressure is modeled by changing the insertion depth of the electrode tip in the tissue. As a consequence, the 6-holes electrode is not considered in this part of the study since the increased insertion depth yields the electrode holes to be no longer located above the cardiac tissue surface, making difficult to estimate the behaviour of the saline flows out of the tip. As Fig 9A shows, all the parameters characterizing the lesion increase with the electrode-tissue contact pressure, regardless of the electrode position. Most important, when the contact pressure changes from 1 to 1.5 mm for both electrode positions, the value of SW shows a significant increase. In the specific, SW grows from 4.5 to 6.2 mm in the perpendicular position, and from 8.5 to 9.7 mm in the parallel position.

Fig 9B shows the corresponding LV and the temperature distributions in the cardiac tissue and blood. LV also grows with the increase of the electrode-tissue contact pressure. In particular, changing the contact pressure from 0.5 to 1.5 mm, LV increases by 144.7 mm^3^ for the electrode in perpendicular position, and by 201.9 mm^3^ for the electrode in parallel position. T_max_blood_ shows a different behavior than LV, where an initial decay is followed by a recovery, although with some differences between electrode positions. In the perpendicular position, the value of T_max_blood_ decreases by 6°C for each further insertion of 0.25 mm between 0.5 mm and 1 mm, then increases by 1°C when the insertion depth is brought to 1.5 mm. In the parallel position, on the other hand, T_max_blood_ initially drops by 5°C when the insertion depth is varied from 0.5 mm to 0.75 mm, remains constant between 0.75 mm and 1 mm, and rises 4°C when the electrode contact pressure is increased further by 0.5 mm. In both cases, however, the value of T_max_blood_ remains below the critical value of 80°C.

## Discussion

We introduce the first complete computational model for an open-irrigated electrode to be used in RFCA. The innovation is to include the interaction between blood motion and saline irrigation, which allows to model realistically not only the geometry of the thermal lesion, but also the maximum temperature reached in the surrounding blood, which is known to have an impact on thrombus formation. The model’s viability was firstly assessed against data from some existing experimental results [3]. Furthermore, we conducted computer simulations under different conditions of irrigation flow rate, electrode positioning (parallel/perpendicular, and tissue insertion depth), and power settings, in order to compare the results to those obtained from other experimental studies in which these issues were assessed, and thus further validate the computer model.

The model was able to adequately reproduce the findings of experimental studies [3,9] showing, as reported, that the electrode design does not significantly affect the lesion volume and depth (see Table 2 and Fig 3). Regarding the effect of the irrigation flow rate, our results (Figs 4 and 5) replicated the performance observed in experiments [7,10,12], i.e. that 1) high flow rates result in lower temperatures at the blood-tissue interface and smaller lesion diameters, 2) lesion depth is not affected by the different irrigation flow rates, and 3) Standard RF applications with open-irrigated electrodes do not reach an interface temperature of 80°C preventing thrombus formation.

As expected [9,29], the computer results (Fig 7) also predicted that deeper and wider lesions are obtained in both perpendicular and parallel positions when a higher RF power is applied.

Regarding the electrode position (parallel vs. perpendicular), our model predicts larger lesion sizes when the electrode position is parallel to the tissue. As of today, the question of which electrode position provides larger lesions is still controversial. In fact, while some experimental studies reported larger lesions with the electrode in parallel contact [3,8–10], others showed smaller lesion sizes with this position [4,27]. Finally, some studies show that the electrode position did not affect to the lesion size [5] or do not find a consistent pattern [26]. It has been suggested that the larger lesions obtained in perpendicular position could be linked with the fact that this positioning would allow for more external irrigation (namely, the holes providing external irrigation are not occluded, as could occur when the tip is parallel to the tissue) [27]. However, our computational model appears to support the findings in other studies [3,8–10] where larger lesions emerge for the parallel position of the catheter, independently of the irrigation flow rate, the applied power and the contact pressure. We think that possibly the discrepancy observed between experimental results about the effect of the electrode positioning could be related to the numerous variables that interplay during an experimental RF application, making the findings difficult to reproduce. In any case, our results support those works that favor the parallel positioning in terms of lesion size.

In our model, the contact pressure was simulated by means of a minimally increasing the depth of the electrode into the tissue. Although it is an artificial solution, the results correlate well with previous published data [10,29], demonstrating a significant increase on lesion volume with the increase of insertion into the tissue, as observed in Fig 9.

### Limitations of the study and future work

We only considered two electrode positions (perpendicular and parallel). These two positions allow a good estimation of the clinical performance of the catheter, even if in clinical practice the catheter position can possibly alternate and feature several intermediate angles between 0–90°. Future work will focused on computer modeling of RFCA at specific locations into the heart. In particular, it will assess which electrode design would be optimal for each ablation by taking into account both anatomical features (which affect the electrode-tissue contact pressure and blood flow direction) and procedural conditions (related with the electrode orientation, applied power and irrigation flow rate). Our validated and accurate computer model makes it possible to test all these conditions easily and reliably, expanding catheter testing beyond the limitations of experimental studies. The conclusions of this future work could propose guidelines to optimize the RFCA in terms of performance and safety.

## Conclusions

We introduced a first computational model for an open-irrigated RFA catheter that takes into account the interaction between blood flow and saline irrigation. The model was successfully validated against previous experimental data. The model shows great potential in developing intervention strategies in terms of electrode design, tuning of the flow rate and the applied RF power to optimize arrhythmia ablation.
